# A unified ab initio theory of spin-phonon relaxation and decoherence uncovers fast dephasing in magnetic molecules

**DOI:** 10.1126/sciadv.aeb3868

**Published:** 2026-03-20

**Authors:** Alessandro Lunghi

**Affiliations:** School of Physics, AMBER and CRANN Institute, Trinity College, Dublin 2, Ireland.

## Abstract

Spin-phonon interactions are known to drive magnetic relaxation in solid-state systems but are generally overlooked as a contribution to spin decoherence through dephasing. Here, we extend quantum master equations to account for coherence terms and describe the full effect of up to two-phonon processes on spin dynamics. We implement this method fully ab initio for a molecule with large magnetization blocking temperature and show that, although strong axial magnetic anisotropy ensures slow magnetic relaxation approaching seconds at 77 kelvins, the superposition of Kramers doublets is coherent for less than 10 nanoseconds due to a two-phonon pure dephasing mechanism. This process, in principle, applies to any quantum system interacting with a thermal bath of phonons, advancing our understanding of quantum decoherence in solid-state systems.

## Teaser

Phonons induce fast dephasing in magnetic molecules.

## INTRODUCTION

The possibility to coherently superimpose observable states of matter is the hallmark of quantum mechanics. Although bewildering, concepts such as quantum coherence and entanglement have enabled fascinating realizations of new physical phenomena and technologies (*1*). The spin of particles like electrons and nuclei holds a special place within this phenomenology and its coherent states are routinely used in magnetic resonance experiments, such as in imaging (*2*) or quantum sensing (*3*).

Coherence is a fragile state of matter, and the interaction between the spin and its environment can quickly lead a quantum state to decay into a mixture of classical ones, a process known as decoherence. The rate of decoherence rate, $T_{2}^{-1}$, is often described in terms of two other time constants

$$\frac{1}{T_{2}}=\frac{1}{2T_{1}}+\frac{1}{T_{2}^{*}}$$ (1)

where $T_{1}$ is referred to as the spin relaxation time, and $T_{2}^{*}$ is referred to as the pure dephasing time. These two time constants can be measured directly in molecules with unpaired electrons, and several studies have been done to understand their microscopic origin. Two very different physical mechanisms are generally assumed to underpin relaxation and dephasing. In the first case, the exchange of energy with the lattice through spin-phonon interactions is deemed responsible, whereas in the second case, dephasing is assumed to occur through a time-dependent magnetic noise generated by pairs of spins in the environment simultaneously flipping their orientation (*4*).

Electron paramagnetic resonance of 3d ions generally shows that, when temperatures are low enough, relaxation time becomes very long, leaving spin-spin dipolar interactions to limit coherence time under such conditions (*5*–*7*). However, dipolar contributions to $T_{2}$ can be mitigated by deuteration and using proton-free environments (*8*) or through exploiting clock transitions (*9*, *10*), suggesting that spin relaxation would remain the hard limit for $T_{2}$ at all temperatures, but cryogenic ones. Moreover, dynamical decoupling, an experiment where a series of microwave pulses are used to refocus the precession of spin, can be used to remove the contribution of pure dephasing (*11*). According to Eq. 1, under such conditions, one would expect to obtain a value of $T_{2}$ matching the limit of $2T_{1}$. On the other hand, experimental evidence has shown that this does not necessarily happen, and a gap may remain between $T_{2}$ and $2T_{1}$ (*11*, *12*). A similar scenario seems to be emerging from the study of clock transitions, where magnetic noise other than the dipolar one limits $T_{2}$ (*13*). Both simulations (*14*, *15*) and experiments (*16*) have recently advanced the hypothesis that a vibrational contribution to $T_{2}^{*}$ might be operative, possibly explaining this experimental evidence, but a comprehensive theory of such a fundamental process is still missing.

Here, we address this knowledge gap on such a fundamental physical process by tapping into the increasingly successful field of ab initio spin dynamics. This method has already provided unique insights into the spin-phonon relaxation of magnetic molecules, ranging from isotropic spin-1/2 systems (*17*) up to strongly correlated 3d metal (*18*, *19*) or lanthanide complexes (*20*, *21*) and solid-state paramagnetic defects (*15*, *22*). A theory of spin-phonon relaxation up to two-phonon contributions has thus far been developed (*17*, *18*). However, whereas second-order contributions have been fully unraveled (*23*, *24*), fourth-order contributions, key to describing spin relaxation across all kinds of molecular systems (*17*, *18*), have thus far been determined only for the population terms of the density matrix (*18*). In addition to precluding gaining insights on coherence times, this limitation has been shown to potentially lead to catastrophic results in the case of spectral degeneracies (*18*).

Here, we extend the formalism of fourth-order ab initio spin relaxation theory to the calculation of decoherence and apply it to a paradigmatic molecule with long magnetization relaxation times. The study of this class of systems, also known as single-molecule magnets (SMMs), has particularly enjoyed large attention in recent years thanks to the possibility of chemically tailoring their magnetic anisotropy. In particular, for the case of coordination compounds based on Dy^3+^ ions with large effective total magnetic moment J=15/2 and magnetization reversal barriers exceeding 2000 K (*20*, *25*, *26*), record-breaking long magnetization relaxation times, τ, have been achieved. The application of SMMs in quantum information science is also being heavily pursued due to several potential advantageous properties of this physical platform, including chemical and physical tunability, synthetic reproducibility, and more (*27*, *28*). Therefore, the application of the proposed ab initio theory of spin-phonon decoherence to these systems has a twofold value. On the one hand, it serves as a benchmark system to illustrate the nature of a fundamental process such as phonon decoherence. On the other hand, it provides unprecedented insights into the effects of phonons on determining the ultimate limit to spin coherence time in solid-state molecular systems, thus determining how far this physical platform can be expected to contribute to quantum science and technology.

Results show that a pure-dephasing mechanism of decoherence, purely of phononic nature, emerges at the fourth order of theory. In the case of SMMs with large magnetic anisotropy, this contribution remains extremely efficient despite the engineering of the ion’s crystal field and spin-phonon interactions and undercuts the classical magnetization reversal time by eight orders of magnitude. This process is expected to be universal and in principle applies to any quantum system coupled to a bosonic heat reservoir, therefore marking a key step forward in understanding the microscopic origin of quantum decoherence in solid-state systems.

## RESULTS

### Quantum master equations

To access coherence time over the full temperature range, we here set out to derive a fourth-order generator able to describe the time evolution of the entire spin density matrix.

Let us start by describing the system Hamiltonian as

$$\hat{H}={\hat{H}}_{s}+{\hat{H}}_{\text{ph}}+{\hat{H}}_{\text{sph}}$$ (2)

where ${\hat{H}}_{s}$ is the spin Hamiltonian, and ${\hat{H}}_{\text{ph}}$ is the phonon Hamiltonian. The latter is assumed to be harmonic

$${\hat{H}}_{\text{ph}}=\sum_{\alpha }\hbar \omega_{\alpha }\left({\hat{n}}_{\alpha }+\frac{1}{2}\right)$$ (3)

where $\hbar \omega_{\alpha }$ is the α-phonon quantum energy and ${\hat{n}}_{\alpha }={\hat{a}}_{\alpha }^{†}{\hat{a}}_{\alpha }$ is the number operator written in terms of creation and annihilation operators. Last, the third term in Eq. 2 is the spin-phonon interactions, which in the weak-coupling linear regime reads

$${\hat{H}}_{\text{sph}}=\sum_{\alpha }\left(\frac{\partial {\hat{H}}_{s}}{\partial q_{\alpha }}\right){\hat{q}}_{\alpha }$$ (4)

where ${\hat{q}}_{\alpha }=\left({\hat{a}}_{\alpha }^{†}+{\hat{a}}_{\alpha }\right)$ are the phonons’ atomic displacement operators. The canonical 2^–1/2^ factor appearing in the definition of ${\hat{q}}_{\alpha }$ is here absorbed into the derivative term of Eq. 4.

The time evolution of the spin system under the influence of a weakly coupled phonon bath is here tackled with time-local quantum master equations (*29*)

$$\frac{d\hat{\rho }\left(t\right)}{\mathrm{dt}}=\hat{\hat{R}}\left(t,t_{0}\right)\;\hat{\rho }\left(t\right)\overset{t_{0}\to -\infty }{\to }\hat{\hat{R}}\;\hat{\rho }\left(t\right)$$ (5)

where $\hat{\hat{R}}$ is a superoperator describing the effect of the phonon bath over the dynamics of the reduced spin density operator $\hat{\rho }\left(t\right)$ expressed in the interaction picture. As depicted in Eq. 5, the superoperator $\hat{\hat{R}}$ becomes time independent in the Markovian limit, i.e., long times after the system has been initialized at $t_{0}$. The latter approximation is well justified for molecular crystals as phonons relax to equilibrium on much faster timescales than spin (*30*, *31*). To make Eq. 5 tractable, the superoperator $\hat{\hat{R}}$ is often expanded perturbatively, $\hat{\hat{R}}=\sum_{n=1}^{\infty }{\hat{\hat{R}}}^{\left(2n\right)}$, and recently the regularized T-matrix approach (*29*) has been used to determine an explicit expression of ${\hat{\hat{R}}}^{\left(2\right)}$ and ${\hat{\hat{R}}}^{\left(4\right)}$, driving the population terms of $\hat{\rho }$ under the Markov and secular approximations (*18*, *23*). Here, we extend the same formalism to the full reduced spin density matrix up to the fourth order and report the full derivation and expression in the Supplementary Materials. To easily visualize the mathematical structure of these equations, we recall that any process described by a Markovian semigroup leads to a generalized and universal Lindblad form of quantum master equations (*32*), which, once expressed in the eigenbasis of ${\hat{H}}_{s}$, read

$$\begin{array}{c} {\dot{\rho }}_{\mathrm{ab}}=\sum_{\mathrm{cd}}R_{\mathrm{ab},\mathrm{cd}}\;\rho_{\mathrm{cd}}\left(t\right)=\\ \sum_{\mathrm{cd}}\sum_{\kappa }\gamma_{\kappa }\left[{\left(L_{\mathrm{bd}}^{\kappa }\right)}^{*}L_{\mathrm{ac}}^{\kappa }-\frac{\delta_{\mathrm{bd}}}{2}\sum_{e}{\left(L_{\mathrm{ea}}^{\kappa }\right)}^{*}L_{\mathrm{ec}}^{\kappa }-\frac{\delta_{\mathrm{ac}}}{2}\sum_{e}{\left(L_{\mathrm{ed}}^{\kappa }\right)}^{*}L_{\mathrm{eb}}^{\kappa }\right]\rho_{\mathrm{cd}}\left(t\right) \end{array}$$ (6)

where the jump operators ${\hat{L}}_{\kappa }$ represent the effect of the bath on the reduced density matrix operator, and $\gamma_{\kappa }$ are positive weights determining the relative efficiency of different κ bath contributions. As shown in the Supplementary Materials, it is possible to define jump operators at different orders n and to obtain explicit expressions for $R_{\mathrm{ab},\mathrm{cd}}^{\left(2n\right)}$. Under the secular approximation, i.e., $R_{\mathrm{ab},\mathrm{cd}}=0$ unless $\omega_{\mathrm{db}}=\omega_{\mathrm{ac}}=\omega$, the second-order jump operators read

$$\sqrt{\gamma_{\kappa }}L_{\mathrm{ac}}^{\kappa }=\frac{\sqrt{2\pi \;G^{\left(2\right)}\left(\omega ,\omega_{\alpha }\right)}}{\hbar }V_{\mathrm{ac}}^{\alpha }$$ (7)

where κ=α, $V_{\mathrm{ac}}^{\alpha }=\langle a|\partial {\hat{H}}_{s}/\partial Q_{\alpha }|c\rangle$, and

$$G^{\left(2\right)}\left(\omega ,\omega_{\alpha }\right)=\delta \left(\omega -\omega_{\alpha }\right){\bar{n}}_{\alpha }+\delta \left(\omega +\omega_{\alpha }\right)\left({\bar{n}}_{\alpha }+1\right)$$ (8)

In Eq. 8, ${\bar{n}}_{\alpha }={\left(e^{\hbar \omega_{\alpha }/k_{B}T}-1\right)}^{-1}$ is the Bose-Einstein distribution accounting for the thermal population of phonons, *k*_B_ is the Boltzmann constant, and the Dirac delta functions enforce energy conservation during the absorption and emission of phonons by the spin system, respectively.

The definition of the fourth-order jump operators accounts for all different two-phonon processes that can affect the time evolution of the spin density matrix, namely, the double absorption of phonons, the double emission of phonons, and the absorption of one phonon and simultaneous emission of a second one. The explicit form for the latter contribution reads

$$\sqrt{\gamma_{\kappa }}L_{\mathrm{ac}}^{\kappa }=\frac{\sqrt{2\pi \;G_{-+}^{\left(4\right)}\left(\omega ,\omega_{\alpha },\omega_{\beta }\right)}}{\hbar }\left[T_{\mathrm{ac}}^{\alpha \beta ,+}+T_{\mathrm{ac}}^{\beta \alpha ,-}\right]$$ (9)

where κ needs now to be interpreted as running over any distinct pair of (α,β) phonons, and we have introduced the terms

$$T_{\mathrm{ac}}^{\alpha \beta ,\pm }=\sum_{f}\frac{V_{\mathrm{af}}^{\alpha }V_{\mathrm{fc}}^{\beta }}{E_{f}-E_{c}\pm \hbar \omega_{\beta }}$$ (10)

which describe the contribution of virtual transitions to excited spin states ∣f〉. Last, $G_{-+}^{\left(4\right)}$ reads

$$G_{-+}^{\left(4\right)}\left(\omega ,\omega_{\alpha },\omega_{\beta }\right)=\delta \left(\omega -\omega_{\alpha }+\omega_{\beta }\right){\bar{n}}_{\alpha }\left({\bar{n}}_{\beta }+1\right)$$ (11)

The full expression of the fourth-order jump operators is reported in the Supplementary Materials. Equations like Eqs. 7 and 9 can then be used to populate Eq. 6 and describe the dynamics of the full spin density matrix, which was previously limited to the second order. For completeness, we also mention that it is, in principle, possible to extend the interaction Hamiltonian in Eq. 4 to account for quadratic coupling (*24*). In that case, an extra two-phonon contribution to both population and coherence dynamics arises, as previously reported (*14*, *15*). This contribution should also be accounted for in the general case but will not be considered here as it is not expected to be relevant for the specific molecule addressed in this work (vide infra).

### Numerical simulations

**Fig. 1.**
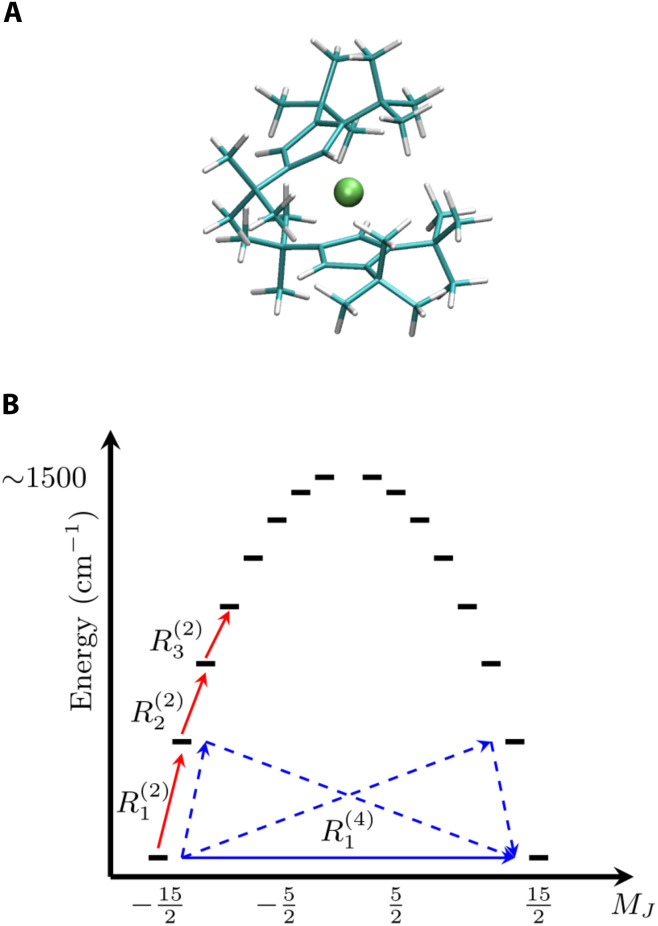
Molecular structure and spin energy levels. (**A**) DyCp molecular structure. Dy is represented in acid green, C is represented in green, and H is represented in white. (**B**) The Orbach relaxation process is depicted with red continuous arrows, each mediated by a second-order transition with rate $R_{i}^{\left(2\right)}$. The continuous blue arrow represents the fourth-order transition with rate $R_{1}^{\left(4\right)}$ causing Raman relaxation. The dashed blue arrows show the virtual transitions.

We numerically implement the calculation of $R_{\mathrm{ab},\mathrm{cd}}^{\left(2\right)}$ and $R_{\mathrm{ab},\mathrm{cd}}^{\left(4\right)}$ for the SMM [${\text{DyCp}}_{2}^{\text{ttt}}$]^+^ (Cp^ttt^=[C_5_H_5_*^t^*Bu_3_-1,2,4]), DyCp in short from now on, a coordination complex exhibiting a J=15/2 ground state and a record-breaking zero-field splitting of the *M_J_* components of about 1500 cm^−1^ (*20*), as depicted in Fig. 1.

**Fig. 2.**
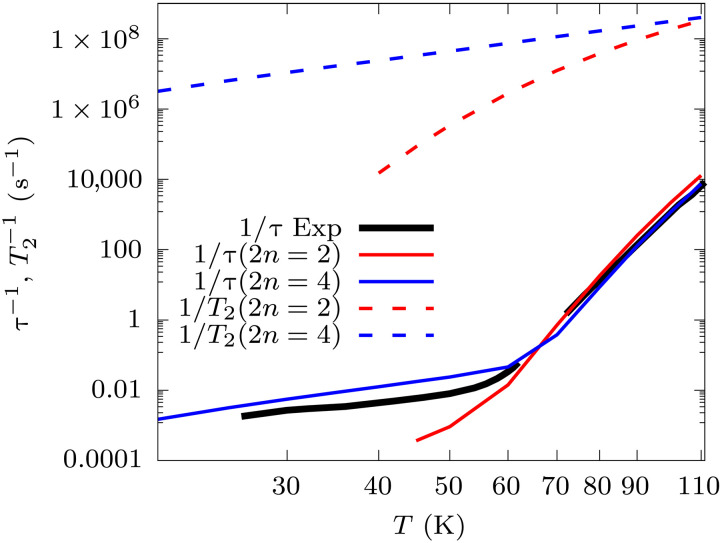
Magnetic relaxation and decoherence. The experimental magnetization relaxation time (*20*) is reported with a continuous black line. Simulated magnetic relaxation is reported in continuous lines. Simulated decoherence time for the ground state KD is reported with dashed lines. Second-order results are in red, and fourth-order ones are in blue.

The spin Hamiltonian of this compound can be modelled through a zero-filed splitting term expressed as a combination of tesseral operators and a Zeeman term

$${\hat{H}}_{s}=\sum_{l=2}^{6}\sum_{m=-l}^{l}B_{m}^{l}{\hat{O}}_{m}^{l}\left(\vec{J}\right)+\mu_{B}g_{J}\vec{J}\cdot \vec{B}$$ (12)

with *l* restricted to even values to preserve Kramers degeneracy in the absence of magnetic fields, *g_J_* is the Lande factor for J=15/2 and $\mu_{B}$ is the bohr magneton. Unless specified otherwise, all simulations are conducted by orienting the easy axis of ground-state Kramers doublets (KDs) along *z* and applying a magnetic field of 1000 Oe, as often done in experiments. DyCp’s crystal phonons, the coefficients $B_{\mathrm{lm}}$, and their derivatives fully define Eq. 2 and have been previously computed through a combination of density functional theory and multireference quantum chemistry (*18*). Values of magnetic relaxation rate, $\tau^{-1}$, are extracted from simulations by diagonalizing the generalized matrices ${\hat{\hat{R}}}^{\left(2n\right)}$ and identifying the eigenvalue associated to an eigenvector corresponding to a transition between the states forming the fundamental KD, i.e., the states with maximum and minimum magnetization value. The latter usually corresponds to the first nonzero eigenvalue of ${\hat{\hat{R}}}^{\left(2n\right)}$. Notably, we show in the Supplementary Materials that such a simulation of magnetic relaxation time can now be obtained without the need to break Kramers degeneracy, e.g., by introducing an external magnetic field, and results are fully rotationally invariant, overcoming previous theoretical limitations (*18*). This is guaranteed by the fact that we are now computing all secular matrix elements of $R_{\mathrm{ab},\mathrm{cd}}^{\left(4\right)}$, instead of restricting ourselves to the diagonal ones $R_{\mathrm{aa},\mathrm{bb}}^{\left(4\right)}$. Similar to what was observed previously at the second order (*18*), we here numerically find that the eigenvalues of $R_{\mathrm{ab},\mathrm{cd}}^{\left(4\right)}$ are invariant with respect to molecular orientation in zero field. Simulations results, reported in Fig. 2, accurately reproduce experiments across the entire available dataset. This observation supports the assumption that linear spin-phonon coupling is the only relevant interaction for this particular system, and we leave the exploration of quadratic coupling, not deemed here essential, to future work. Above about 60 K, ${\hat{\hat{R}}}^{\left(2\right)}$ determines the relaxation of the magnetization through the Orbach mechanism, which involves subsequent single-phonon transitions to higher energy KDs (schematically depicted in Fig. 1) and eventually reaching the reversal of the magnetization by emitting single-phonons back to the ground-state KD. At low temperature, the effect of ${\hat{\hat{R}}}^{\left(4\right)}$ leads to two-phonon Raman relaxation, which involves an intra–ground-state KD process mediated by virtual transitions to excited KDs and the simultaneous emission and absorption of a pair of phonons whose energy difference matches the Zeeman splitting of the ground-state KD.

We then turn to the simulation of coherence times, now enabled by the full expression of ${\hat{\hat{R}}}^{\left(2\right)}$ and ${\hat{\hat{R}}}^{\left(4\right)}$. To do this, we choose to study the coherent superposition among the ground-state KD, namely, between the states $|a\rangle =|M_{J}≃15/2\rangle$ and $|b\rangle =|M_{J}≃15/2\rangle$. Figure 2 reports the values of *T*_2_, computed as $T_{2}^{-1}=-R_{\mathrm{ab},\mathrm{ab}}^{\left(2n\right)}$. The latter equality holds as long as no degeneracies are present for the states *a* and *b*; otherwise, a full determination of the dynamics of $\rho_{\mathrm{ab}}\left(t\right)$ should be performed. Simulated coherence times are markedly shorter than the relaxation time of the molecular magnetization. These computed values of *T*_2_ closely resemble the catastrophic values of τ previously computed through the diagonalization of $R_{\mathrm{aa},\mathrm{bb}}^{\left(4\right)}$ without taking care of orienting the molecule along its magnetic easy axis and applying an external field. This result is purely coincidental and stems from the fact that the matrix elements $R_{\mathrm{ab},\mathrm{ab}}^{\left(4\right)}$ for the molecule oriented along the easy axis get rotated into $R_{\mathrm{aa},\mathrm{bb}}^{\left(4\right)}$ for the specific orientation of the molecule in the crystal. This fact, in addition to truncating the full $R_{\mathrm{ab},\mathrm{cd}}^{\left(4\right)}$ operator to its diagonal form, led to the wrong interpretation of fast decoherence as fast magnetic relaxation. Now that we have access to the full fourth-order master matrix, these ambiguities are lifted, and we are in the position to correctly assign the right values to different processes as well as study their orientation and field dependence.

To interpret the origin of such a short coherence time, we start by writing down the expression of *T*_2_ for a pair of states. As detailed in the Supplementary Materials, it follows from Eq. 6 that the spin relaxation and pure spin dephasing contributions to decoherence can be expressed as

$$\frac{1}{2T_{1}}=\sum_{\kappa }\gamma_{\kappa }\left[\frac{1}{2}\sum_{e\neq a}{\left(L_{\mathrm{ea}}^{k}\right)}^{*}L_{\mathrm{ea}}^{k}+\frac{1}{2}\sum_{e\neq b}{\left(L_{\mathrm{ed}}^{k}\right)}^{*}L_{\mathrm{eb}}^{k}\right]$$ (13)

and

$$\frac{1}{T_{2}^{*}}=\sum_{\kappa }\gamma_{\kappa }\left[-{\left(L_{\mathrm{bb}}^{\kappa }\right)}^{*}L_{\mathrm{aa}}^{\kappa }+\frac{1}{2}{\left(L_{\mathrm{aa}}^{\kappa }\right)}^{*}L_{\mathrm{aa}}^{\kappa }+\frac{1}{2}{\left(L_{\mathrm{bb}}^{\kappa }\right)}^{*}L_{\mathrm{bb}}^{\kappa }\right]$$ (14)

**Fig. 3.**
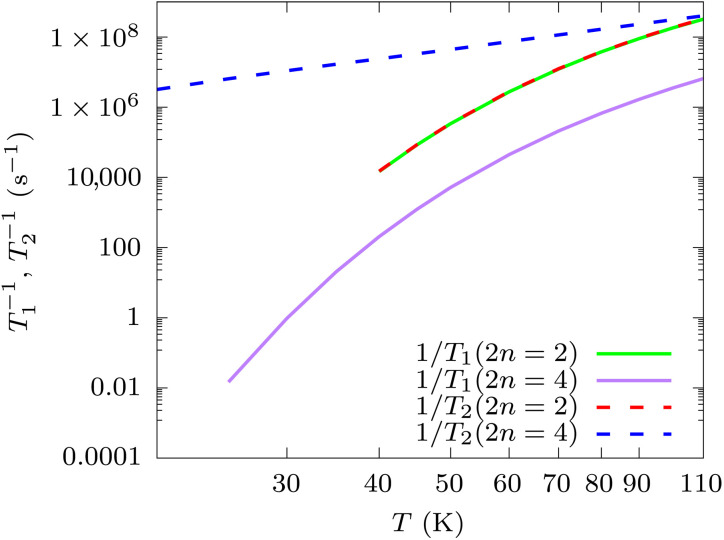
Relaxation and dephasing contributions to decoherence. Second-order (2n=2) $T_{2}$ and $T_{1}$ are reported in continuous green and dashed red lines, respectively. Fourth-order (2n=4) $T_{2}$ and $T_{1}$ are reported in continuous purple and dashed blue lines, respectively. The time constants refer to the coherence superposition of the ground-state KD states.

The first thing to note is that, in multilevel systems, such as DyCp, the magnetic relaxation time, τ, and the *T*_1_ contribution to *T*_2_ for a pair of states might be intrinsically different. For instance, in the high-temperature regime dominated by Orbach relaxation, a series of single-phonon absorptions is necessary to reverse the magnetic moment and cause its relaxation, with the overall rate of the total process diminishing for each additional step taken to climb the magnetization reversal barrier. On the other hand, the *T*_1_ for a pair of states is limited by transitions to any state, as expressed by Eq. 13, not necessarily requiring climbing the full magnetization reversal energy barrier and thus occurring at a much faster rate. This is confirmed by Fig. 3, which shows that *T*_1_ exponentially decreases with temperature, $A\;\text{exp}\left(-U/k_{B}T\right)$, with *U* = 438.0 cm^−1^ and *U* = 466.9 cm^−1^, for the second and fourth order, respectively, and is thus mediated by phonon-induced transitions to the first excited KD computed at about 451 cm^−1^.

**Fig. 4.**
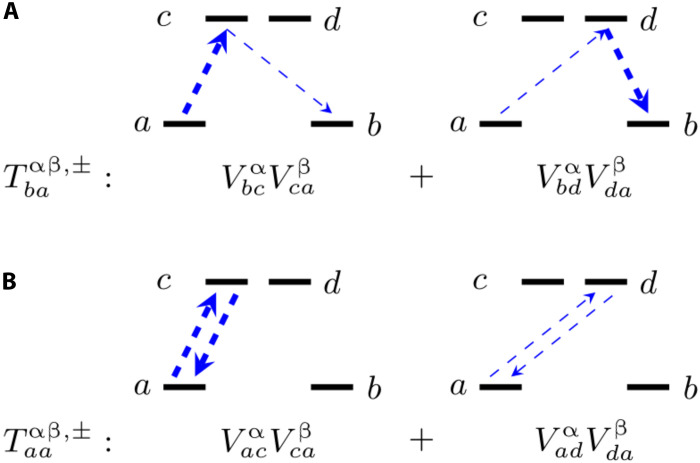
Diagrammatic representation of the virtual transitions. (**A**) Fourth-order relaxation. (**B**) Fourth-order dephasing. Arrow thickness is proportional to the rate of the transitions.

Figure 3 also shows that, at the second order, *T*_2_ and *T*_1_, are identical. This is consistent with a vanishing ${\left(T_{2}^{*}\right)}^{-1}$, being a zero-energy process (ω=0) not achievable by exchanging a single phonon at the time. To visualize this, one can notice that $T_{2}^{*}$ depends on the diagonal elements of the jump operators, e.g., $L_{\mathrm{aa}}^{\kappa }$. At the second order, these introduce a multiplication by the vanishing contribution of $G^{\left(2\right)}\left(\omega_{\mathrm{aa}}=0,\omega_{\alpha }\right)$. At the fourth order, we instead observe that $T_{2}^{*}$ is the limiting factor for $T_{2}$. The large difference between τ and $T_{2}$ can be understood by studying the terms of Eq. 10. In the case of τ, the relevant jump operators’ matrix elements are $L_{\mathrm{ba}}^{\kappa }$, which, at the fourth order, include contributions from $T_{\mathrm{ba}}^{\alpha \beta ,\pm }$. The latter is dominated by virtual transitions to the first excited KD (*18*) mediated by the product of matrix elements $V_{\mathrm{bc}}^{\alpha }V_{\mathrm{ca}}^{\beta }$, as schematically represented in Fig. 4A. The electronic structure of SMMs such DyCp is engineered specifically to make $V_{\mathrm{ca}}$ very small when *c* and *a* are sampled from opposite sides of the magnetization reversal barrier, making magnetic relaxation progressively more inefficient as the KDs get closer to pure *M_J_* states. In the case of fourth-order pure dephasing, however, the relevant terms of jump operators are of the form $L_{\mathrm{aa}}^{\kappa }$ and $T_{\mathrm{aa}}^{\alpha \beta ,\pm }$, which instead include the product of matrix elements $V_{\mathrm{ac}}^{\alpha }V_{\mathrm{ca}}^{\beta }$ (see Fig. 4B). Although this number will be vanishingly small when *a* and *c* correspond to states with *M_J_* of opposite sign, it will be large for transitions to a KD with $\Delta M_{J}=\pm 1$, explaining the efficiency of Raman pure dephasing.

**Fig. 5.**
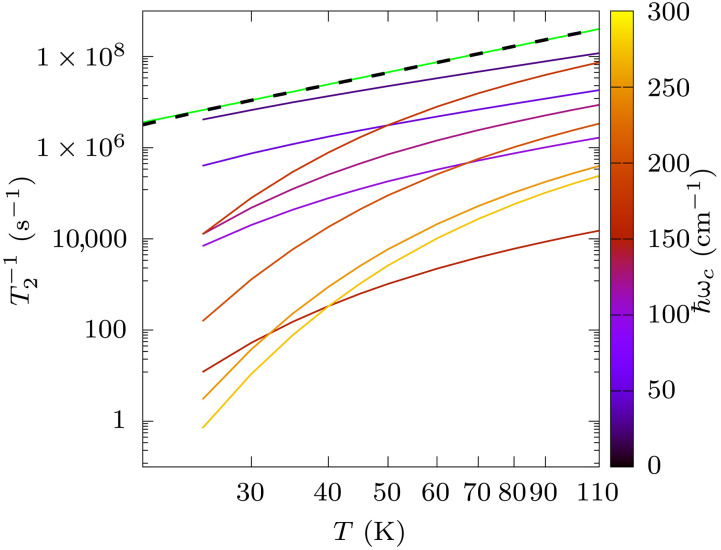
Phonon decomposition of coherence time. The dashed black line corresponds to the total coherence time. The continuous green line is the best fit of the total $T_{2}$ with the function $T_{2}^{-1}=A\;T^{n}$, with *A* = 915.27 (sK)^−1^ and *n* = 2.76. Phonons are grouped in bundles of energy $\hbar \omega_{c}$ + 25 cm^−1^, starting at $\hbar \omega_{c}$ = 25 cm^−1^. Each bundle is used independently to compute $T_{2}$ and reported with a color code indicating $\omega_{c}$.

**Fig. 6.**
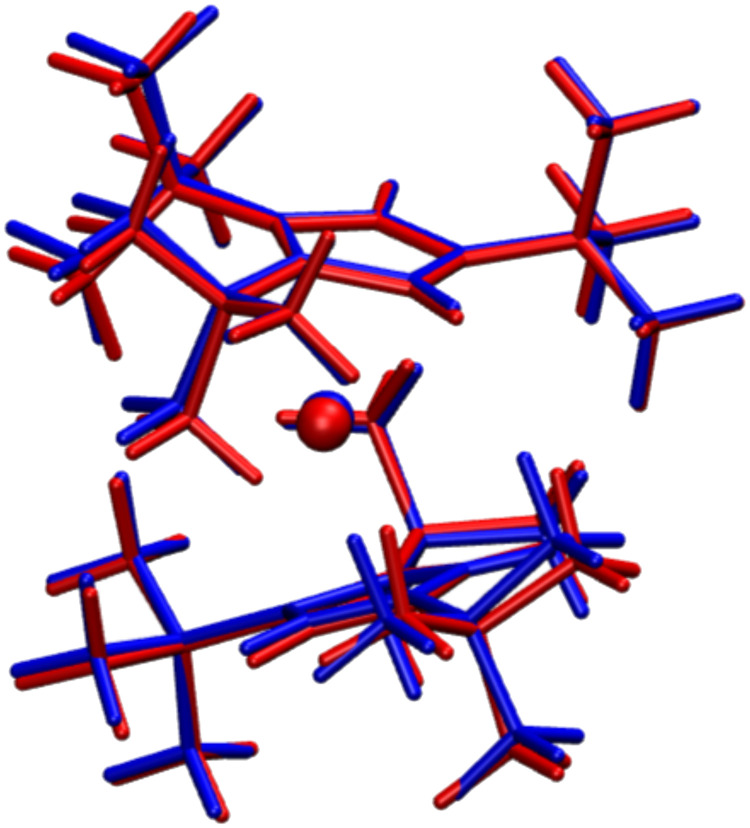
Low-energy phonon displacements. The DyCp equilibrium molecular structure is reported in blue, and the one in red corresponds to the distortion that DyCp undergoes according to the 20th phonon of the computed spectrum with energy ℏω ~ 45 cm^−1^.

Last, we note that the fourth-order contribution to *T*_2_ approximately follows a temperature power law with exponent n=2.76 (see Fig. 5), which is close to the high-*T* limit for a two-phonon process (n=2) mediated by phonons within a small energy window but does not quite match any of the exponents predicted by the canonical theory of spin-lattice relaxation (*33*). We thus investigate further the nature of the phonons contributing to $T_{2}$ by extracting the contribution for phonons at different energies. Figure 5 reports the simulation of $T_{2}$ by including only phonons between a variable cutoff frequency ω*_c_* and ω*_c_* + 25.0 cm^−1^ and shows two main facts. First, at low temperature, decoherence is mostly due to low-energy phonons in the window of 25 to 50 cm^−1^. However, as the temperature increases, modes with higher energies, ~175 to 200 cm^−1^, become thermally activated and contribute equally as much as the low-energy ones to *T*_2_ by the time the temperature approaches 110 K. The visual inspection of the molecular displacements for these two bundles of phonons shows that they all correspond to the motion of the *t*Bu groups decorating the Cp rings, as represented in Fig. 6.

## DISCUSSION

The contribution of phonons to pure dephasing is often overlooked under the general assumption that only spin-spin dipolar interactions are capable of contributing to it. Only recently, second-order spin-phonon contributions to *T*_2_ have been theoretically predicted in spin-1/2 molecules (*14*) and solid-state defects (*15*) and later experimentally observed (*16*), illustrating that spin-phonon coupling contributes to pure dephasing. Here, we have extended the treatment of time-local master equations to the simulation of coherence terms up to the fourth order and achieved a unified picture of spin relaxation and decoherence. On the basis of this theoretical framework, we have identified a phonon pure-dephasing mechanism that arises at the fourth order of theory and is extremely efficient in magnetic anisotropic systems, undercutting the benefit of having a large magnetic anisotropy by several orders of magnitude.

The analysis of the present results also provides some guidelines on how to potentially mitigate dephasing effects. On the basis of Eq. 10, future progress in increasing spin coherence in molecular systems should come from increasing the energy of the lowest-lying KDs and reducing spin-phonon coupling. In this respect, spin-1/2 coordination compounds and organic radicals are the most promising, naturally presenting high-energy electronic excitations and low spin-orbit coupling (*17*, *34*). In addition to this, the analysis of phonon contributions shows that low-energy modes, largely determined by *t*Bu vibrations, are the most responsible for both relaxation and dephasing at low temperature. Embedding molecules into metal-organic or covalent-organic frameworks (*35*, *36*) and rigid cages (*37*) stand out as possible ways forward to stiffen these molecular motions and potentially quench fast dephasing and relaxation. Alternatively, strategies based on the exploration of high-frequency dynamical decoupling (*16*) or the use of the multilevel spin structure typical of high-spin molecular complexes to engineer quantum states resilient to phonon decoherence also appear promising (*38*).

We would like to remark that, despite a direct measurement of *T*_2_ in DyCp or similar systems not yet being available, our predictions are at least indirectly validated by accurately reproducing magnetic relaxation data. The ingredients entering the simulation of the latter are exactly the same used to compute $T_{2}^{*}$, thus offering support to the quality of our predictions. Looking forward to an experimental validation of the present results, we recognize that it might be challenging due to the vanishing matrix elements of a transverse magnetic field among pure *M_J_* states. However, continuous-wave electron paramagnetic resonance spectra for such highly axial and anisotropic Dy SMMs have nonetheless been reported (*39*), suggesting that our numerical predictions could be experimentally verified. In addition, attempts to measure coherence times in Dy coordination complexes have recently been attempted (*40*), placing them below ~0.1 μs at 4 K, in qualitative agreement with our current finding of very fast dephasing. The present results are expected to be general, and we expect any spin system affected by two-phonon processes to present a qualitatively similar phenomenology, thus greatly expanding the pool of compounds that can be used to explore phononic pure dephasing. In this regard, we note that there have been a number of experimental reports in recent years where deviations between *T*_2_ and $2T_{1}$ are observed to persist at high temperature (*41*) or even in the presence of dynamical decoupling (*36*) and at clock transitions (*13*), where phonons should be the only relevant contribution to *T*_2_, leaving space for a pure-dephasing phonon process as the one described here to take place. Although the contribution of $T_{2}^{*}$ in these systems might not be as large as in DyCp, they could represent better candidates to directly test the theory presented here through experiments, and we hope that these results will elicit a more systematic exploration of vibrational pure-dephasing processes in magnetic molecules.

In conclusion, we have derived a general set of equations describing relaxation and decoherence of any quantum system coupled to a Markovian harmonic bath up to the fourth-order level of perturbation theory. On the one hand, this formalism has uncovered a pure dephasing mechanism markedly affecting spin coherence in magnetic molecules. On the other hand, this formalism can be readily applied to the study of decoherence in several other quantum systems, including spins and electrons in condensed matter or cavities, therefore paving the way to a first-principles description of this fundamental quantum process.
