# Supplementary material for: A unified ab initio theory of spin-phonon relaxation and decoherence uncovers fast dephasing in magnetic molecules
Source: Sci Adv. 2026 Mar 20;12(12):eaeb3868. doi: 10.1126/sciadv.aeb3868 (PMC13004027; doi:10.1126/sciadv.aeb3868)
Supplement: Supplementary file 1 — Supplementary Text Fig. S1 [file sciadv.aeb3868_sm.pdf]

Science Advances
AAAS

Supplementary Materials for

# A unified ab initio theory of spin-phonon relaxation and decoherence uncovers fast dephasing in magnetic molecules

Alessandro Lunghi

Corresponding author: Alessandro Lunghi, lunghia@tcd.ie

*Sci. Adv.* **12**, eaeb3868 (2026)
DOI: 10.1126/sciadv.aeb3868

**This PDF file includes:**

Supplementary Text
Fig. S1

## Derivation of the full 4th-order quantum master equations within the regularized $\mathcal{T}$-matrix formalism

### $\mathcal{T}$-matrix formalism for spin-phonon coupling

Let us consider a generic Hamiltonian

$$\hat{H} = \hat{H}_0 + \hat{V} \ , \tag{S1}$$

where $\hat{V}$ acts as a perturbation to $\hat{H}_0$, and that the spectrum of $\hat{H}_0$ is fully known. At the lowest order of time-dependent perturbation theory ($n = 2$), the evolution of the density matrix of a quantum system, expressed in the interaction picture, can be written as [23]

$$\dot{\rho}_{ab} = \sum_{cd} R^{(2)}_{ab,cd} \rho_{cd}(t) \ , \tag{S2}$$

where

$$R^{(2)}_{ab,cd} = \frac{2\pi}{\hbar} \left[ V_{ac} V^*_{bd}\, \delta(E_a - E_c) - \frac{\delta_{db}}{2} \sum_e V^*_{ea} V_{ec}\, \delta(E_e - E_c) - \frac{\delta_{ac}}{2} \sum_e V^*_{ed} V_{eb}\, \delta(E_e - E_d) \right] \ , \tag{S3}$$

and $V_{ab}$ are the matrix elements of $\hat{V}$ in the representation of the eigenvectors of $\hat{H}_0$ and $E_a$ are the eigenvalues of $\hat{H}_0$. Eqs. (S2-S3) are derived under the Born-Markov and Secular approximations and therefore include the assumption that all terms of $R^{(2)}_{ab,cd}$ vanish unless $\omega = \omega_{ac} = \omega_{db}$.

To go beyond the second-order time-dependent approximation used to derive Eq. (S3), we here introduce an effective Hamiltonian [29],

$$\hat{H}_{\text{eff}} = \hat{H}_0 + \hat{\mathcal{T}} \ , \tag{S4}$$

where the perturbation is not just the bare operator $\hat{V}$, but the operator

$$\hat{\mathcal{T}} |i\rangle = \sum_{\mu=1}^{\infty} \hat{V} \left( \frac{1}{E_i - H_0 + i0^+} \hat{V} \right)^{\mu-1} |i\rangle \ , \tag{S5}$$

which introduces higher-order processes into the time evolution of $\hat{\rho}(t)$ giving origin at a series expansion of $R^{(n)}_{ab,cd}$ as function of $\mu$, namely

$$R_{ab,cd} = \frac{2\pi}{\hbar} \left[ \mathcal{T}_{ac} \mathcal{T}^*_{bd}\, \delta(E_a - E_c) - \frac{\delta_{db}}{2} \sum_e \mathcal{T}^*_{ea} \mathcal{T}_{ec}\, \delta(E_e - E_c) - \frac{\delta_{ac}}{2} \sum_e \mathcal{T}^*_{ed} \mathcal{T}_{eb}\, \delta(E_e - E_d) \right] \ . \tag{S6}$$

For $\mu = 1$, the canonical second-order contribution $R^{(2)}_{ab,cd}$ of Eq. (S3) is obtained.

Let us now specialize these expressions to the case of spin-phonon dynamics. To start we define the Hamiltonian of the total system,

$$\hat{H} = \hat{H}_0 + \hat{V} = \hat{H}_{\text{s}} + \hat{H}_{\text{ph}} + \hat{H}_{\text{sph}} \ , \tag{S7}$$

where the sum of the spin Hamiltonian, $\hat{H}_{\text{s}}$, and the phonons Hamiltonian, $\hat{H}_{\text{ph}}$, corresponds to the unperturbed system and the spin-phonon Hamiltonian, $\hat{H}_{\text{sph}}$, to the perturbation. We will refer to the eigenstates of $\hat{H}_{\text{s}}$ as $|a\rangle$ and to the eigenstates of $\hat{H}_{\text{ph}}$ as $|v\rangle$. Each state $|v\rangle$ will correspond to any of the possible occupations $|n_\alpha, n_\beta, ...\rangle$ of each vibrational degree of freedom. In this notation, the eigenstates of the total $\hat{H}_0$ are indicated as $|i\rangle = |av\rangle$. In this work we limit ourselves to linear spin-phonon coupling, which reads

$$\hat{H}_{\text{sph}} = \sum_\alpha \left( \frac{\partial \hat{H}_{\text{s}}}{\partial q_\alpha} \right) q_\alpha = \sum_\alpha \hat{V}^\alpha q_\alpha \ . \tag{S8}$$

In Eq. (S8), $\hat{q}_\alpha = (a^\dagger_\alpha + a_\alpha)$ are the phonons operators. Since the present work deals with phonons at the sole reciprocal space point $\Gamma$, we present all equations by dropping the reciprocal space index. This is done to keep the notation a bit lighter, but the theory naturally extends to all Brillouin zone points. The derivatives of $\hat{H}_s$ are conventionally computed as

$$\left(\frac{\partial \hat{H}_\mathrm{s}}{\partial q_\alpha}\right) = \sum_i^{3N} \sqrt{\frac{\hbar}{2m_i\omega_\alpha}} L_{i\alpha} \left(\frac{\partial \hat{H}_\mathrm{s}}{\partial X_i}\right) , \tag{S9}$$

where the $X_i$ is the coordinate of $i$-th degree of freedom of the molecular crystal's unit cell and $L_{\alpha i}$ are the eigenvectors of the Hessian matrix of the same. We also note that Eq. (S9) absorbs the $2^{-1/2}$ factor that conventionally multiplies the creation/annihilation operators in the definition of $q_\alpha$.

Before turning to the calculation of $R_{ab,cd}$, we note that the choice of $\hat{H}_0$ implies defining a density matrix that spans both the spin and phonons Hilbert spaces

$$\rho_{ij} = \rho_{a(v)j(w)} \,. \tag{S10}$$

We are however only interested in studying the time evolution of the spin system under the influence of a thermalized Markovian phonon bath, and we therefore define a reduced spin density matrix

$$\hat{\rho}_\mathrm{s} = \mathrm{Tr}_\mathrm{B}(\hat{\rho}_\mathrm{s} \otimes \hat{\rho}^\mathrm{eq}_\mathrm{ph}) \,, \tag{S11}$$

where $\hat{\rho}^\mathrm{eq}_\mathrm{ph}$ represents a canonical thermal density matrix for the phonons and the trace $\mathrm{Tr}_\mathrm{B}$ is taken over the Hilbert space of the phonons. Unless specified otherwise, from now on we will drop the subscript s and use $\hat{\rho}$ to refer to the reduced spin density matrix, while the density matrix with the superscript eq is understood to refer to the sole phonons. As a consequence of these approximations, the equation we seek to derive is in the form

$$\sum_v \dot{\rho}_{ab}\rho^\mathrm{eq}_{vv} = \sum_v \sum_{cd} \sum_w R_{a(v)b(v),c(w)d(w)}\, \rho_{cd}(t)\rho^\mathrm{eq}_{ww} \,, \tag{S12}$$

leading to

$$\dot{\rho}_{ab} = \sum_{cd} \sum_{vw} R_{a(v)b(v),c(w)d(w)}\, \rho_{cd}(t)\rho^\mathrm{eq}_{ww} = \sum_{cd} R_{ab,cd}\, \rho_{cd}(t) \,, \tag{S13}$$

with

$$R_{ab,cd} = \sum_{vw} R_{a(v)b(v),c(w)d(w)}\, \rho^\mathrm{eq}_{ww} \,. \tag{S14}$$

To conclude let us write $R_{ab,cd}$ explicitly with the final notation for the indexes

$$\begin{aligned} R_{ab,cd} = \frac{2\pi}{\hbar} \sum_{vw} \Big[ & \mathcal{T}_{a(v)c(w)} \mathcal{T}^*_{b(v)d(w)}\, \delta(E_a + E_v - E_c - E_w) \\ & - \frac{\delta_{db}\delta_{wv}}{2} \sum_{ex} \mathcal{T}^*_{e(x)a(v)} \mathcal{T}_{e(x)c(w)}\, \delta(E_e + E_x - E_c - E_w) \\ & - \frac{\delta_{ac}\delta_{vw}}{2} \sum_{ex} \mathcal{T}^*_{e(x)d(w)} \mathcal{T}_{e(x)b(v)}\, \delta(E_e + E_x - E_d - E_w) \Big] \rho^\mathrm{eq}_{ww} \,. \end{aligned} \tag{S15}$$

We are now ready to compute the matrix elements of $\hat{\mathcal{T}}$ for increasing values of $\mu$ and introduce them into Eq. (S15) to explicitly determine an expansion of the total $R_{ab,cd}$ to various orders $(n)$, which read

$$R_{ab,cd} = \sum_n R^{(2n)}_{ab,cd} = \sum_n \sum_{vw} R^{(2n)}_{a(v)b(v),c(w)d(w)}\, \rho^\mathrm{eq}_{ww} \,, \tag{S16}$$

where we have pre-empted the result that only even orders will contribute to the final result.

**Second-order quantum master equations**

For $\mu = 1$, the bare spin-phonon coupling operator acts as the perturbation. The first term of Eq. (S15) explicitly reads

$$\sum_{wv} \mathcal{T}_{a(v)c(w)} \mathcal{T}^*_{b(v)d(w)} \, \delta(E_a + E_v - E_c - E_w) \rho^{eq}_{ww} = \\ \sum_{wv} \sum_{\alpha\beta} V^{\alpha}_{ac} (V^{\beta}_{bd})^* \langle v|q_\alpha|w\rangle \langle v|q_\beta|w\rangle^* \delta(E_a + E_v - E_c - E_w) \rho^{\text{eq}}_{ww} \,. \tag{S17}$$

We note that the latter expression is non-zero only if $\alpha = \beta$ and if $|v\rangle$ and $|w\rangle$ differ by no more than one $\alpha$-phonon excitation, i.e. if we specify $|w\rangle = |n_\alpha\rangle$, then $|v\rangle$ must be $|n_\alpha \pm 1\rangle$. For any generic $|w\rangle = |n_\alpha\rangle$, we thus get to

$$\sum_{vw} \mathcal{T}_{a(v)c(w)} \mathcal{T}^*_{b(v)d(w)} \, \delta(E_a + E_v - E_c - E_w) \rho^{\text{eq}}_{ww} = \\ \frac{1}{2} \sum_{w} \sum_{\alpha} V^{\alpha}_{ac} (V^{\alpha}_{bd})^* \Big[ n_\alpha \, \delta(E_a - E_c - \hbar\omega_\alpha) + (n_\alpha + 1) \, \delta(E_a - E_c + \hbar\omega_\alpha) \Big] \rho^{\text{eq}}_{ww} = \\ \frac{1}{2} \sum_{\alpha} V^{\alpha}_{ac} (V^{\alpha}_{bd})^* \Big[ \bar{n}_\alpha \, \delta(E_a - E_c - \hbar\omega_\alpha) + (\bar{n}_\alpha + 1) \, \delta(E_a - E_c + \hbar\omega_\alpha) \Big] \,, \tag{S18}$$

where in the last step we have assumed that phononic states are populated according to the canonical distribution and $\bar{n}_\alpha$ is the Bose-Einstein distribution for a mode with energy $\hbar\omega_\alpha$.

Following analogous steps for the second term of Eq. (S15), we can derive

$$- \frac{\delta_{bd}}{2} \sum_{w} \sum_{ex} \mathcal{T}^*_{e(x)a(w)} \mathcal{T}_{e(x)c(w)} \, \delta(E_e + E_x - E_c - E_w) \rho^{\text{eq}}_{ww} = \\ - \frac{\delta_{bd}}{2} \sum_{e} \sum_{\alpha} (V^{\alpha}_{ea})^* V^{\alpha}_{ec} \Big[ \bar{n}_\alpha \, \delta(E_e - E_c - \hbar\omega_\alpha) + (\bar{n}_\alpha + 1) \, \delta(E_e - E_c + \hbar\omega_\alpha) \Big] \,, \tag{S19}$$

and analogously for the last term in Eq. (S15)

$$- \frac{\delta_{ac}}{2} \sum_{w} \sum_{ex} \mathcal{T}^*_{e(w)d(x)} \mathcal{T}_{e(w)b(x)} \, \delta(E_e + E_x - E_d - E_w) \rho^{\text{eq}}_{ww} = \\ - \frac{\delta_{ac}}{2} \sum_{e} \sum_{\alpha} (V^{\alpha}_{ed})^* V^{\alpha}_{eb} \Big[ \bar{n}_\alpha \, \delta(E_e - E_d - \hbar\omega_\alpha) + (\bar{n}_\alpha + 1) \, \delta(E_e - E_d + \hbar\omega_\alpha) \Big] \,. \tag{S20}$$

In virtue of the fact that $\mathcal{T}$ enters twice into the definition of each term of $R_{ab,cd}$, $\mu = 1$ generates a second-order contribution to $R_{ab,cd}$, which reads

$$R^{(2)}_{ab,cd} = \frac{2\pi}{\hbar^2} \sum_{\alpha} \Big[ V^{\alpha}_{ac} (V^{\alpha}_{bd})^* \, G^{(2)}(\omega_{ac}, \omega_\alpha) - \frac{\delta_{bd}}{2} \sum_{e} (V^{\alpha}_{ea})^* V^{\alpha}_{ec} \, G^{(2)}(\omega_{ec}, \omega_\alpha) - \frac{\delta_{ac}}{2} \sum_{e} (V^{\alpha}_{ed})^* V^{\alpha}_{eb} \, G^{(2)}(\omega_{ed}, \omega_\alpha) \Big] \,, \tag{S21}$$

where we have introduced

$$G^{(2)}(\omega_{ac}, \omega_\alpha) = \bar{n}_\alpha \, \delta(\omega_{ac} - \omega_\alpha) + (\bar{n}_\alpha + 1) \, \delta(\omega_{ac} + \omega_\alpha) \,. \tag{S22}$$

The latter result is identical to the one previously presented in Ref. [23].

**Fourth-order quantum master equation.** Let us now derive an expression for $\mu = 2$. In this case, the $\hat{\mathcal{T}}$ operator reads

$$\hat{\mathcal{T}}|i\rangle = \Big(\hat{\mathcal{T}}^{(\mu=1)} + \hat{\mathcal{T}}^{(\mu=2)}\Big)|i\rangle = \Big(\hat{H}_{sph} + \hat{H}_{sph}\frac{1}{E_i - \hat{H}_0 + i0^+}\hat{H}_{sph}\Big)|i\rangle\,. \tag{S23}$$

Before proceeding, we note that by introducing this operator into Eq. (S15) would generate four terms, one corresponding to the product of two $\hat{\mathcal{T}}^{(\mu=1)}$ terms, one product of two $\hat{\mathcal{T}}^{(\mu=2)}$ terms and two cross products. It can be shown that within the approximations made, the cross terms vanish. This is because we would be computing terms of the form

$$\langle v|q_\alpha|w\rangle\langle v|q_\beta q_\gamma|w\rangle\,, \tag{S24}$$

which are all equally zero for any combination of $\alpha$, $\beta$, $\gamma$. To see this, one should notice that the first braket changes the population of one phonon mode by 1, while the second braket will change the population of one mode by either 2 or 0, or two modes by 1, but never a single mode by 1. Since the two states $|v\rangle$ and $|w\rangle$ are the same in the two brakets, the latter are never simultaneously different from zero. From this, it follows that the product of two $\hat{\mathcal{T}}^{(\mu=2)}$ will give rise to the next term of the expansion of $R_{ab,cd}$, namely the fourth-order contribution $R^{(4)}_{ab,cd}$.

Let us start by deriving an expression for the matrix elements of $\hat{\mathcal{T}}^{(\mu=2)}$, which read

$$\mathcal{T}_{a(v)c(w)} = \sum_{\alpha\beta}\sum_{fy}\frac{V^\alpha_{af}V^\beta_{fc}}{E_c + E_w - E_f - E_y + i0^+}\langle v|q_\alpha|y\rangle\langle y|q_\beta|w\rangle\,. \tag{S25}$$

Using this expression, we can now write out the first term of Eq. (S15)

$$\sum_{wv}\mathcal{T}_{a(v)c(w)}\mathcal{T}^*_{b(v)d(w)}\delta(E_a + E_v - E_c - E_w)\rho^{\rm eq}_{ww} = \tag{S26}$$

$$\sum_{wv}\sum_{\alpha\beta}\sum_{\gamma\delta}\Big(\sum_{fy}\frac{V^\alpha_{af}V^\beta_{fc}}{E_c + E_w - E_f - E_y + i0^+}\langle v|q_\alpha|y\rangle\langle y|q_\beta|w\rangle\Big)$$
$$\cdot\Big(\sum_{gz}\frac{(V^\gamma_{bg})^*(V^\delta_{gd})^*}{E_d + E_w - E_g - E_z - i0^+}\langle v|q_\gamma|z\rangle^*\langle z|q_\delta|w\rangle^*\Big)\,\delta(E_a + E_v - E_c - E_w)\rho^{\rm eq}_{ww}\,. \tag{S27}$$

Let us now focus on the phonons' matrix elements and assume without any loss of generality that $|w\rangle = |n_\alpha n_\beta\rangle$. Let us start by considering the $\alpha \neq \beta$ case and we will come back to the $\alpha = \beta$ case at the end. The matrix elements in Eq. (S27) will not vanish only if $(\alpha,\beta) = (\gamma,\delta)$ or $(\alpha,\beta) = (\delta,\gamma)$ or $(\alpha,\gamma) = (\beta,\delta)$, leading to

$$\sum_{vw}\sum_{\alpha\beta}\Big[\Big(\sum_{fy}\frac{V^\alpha_{af}V^\beta_{fc}}{E_c + E_w - E_f - E_y + i0^+}\langle v|q_\alpha|y\rangle\langle y|q_\beta|w\rangle\Big)$$
$$\cdot\Big(\sum_{gz}\frac{(V^\alpha_{bg})^*(V^\beta_{gd})^*}{E_d + E_w - E_g - E_z - i0^+}\langle v|q_\alpha|z\rangle^*\langle z|q_\beta|w\rangle^*\Big)$$
$$+\Big(\sum_{fy}\frac{V^\alpha_{af}V^\beta_{fc}}{E_c + E_w - E_f - E_y + i0^+}\langle v|q_\alpha|y\rangle\langle y|q_\beta|w\rangle\Big)$$
$$\cdot\Big(\sum_{gz}\frac{(V^\beta_{bg})^*(V^\alpha_{gd})^*}{E_d + E_w - E_g - E_z - i0^+}\langle v|q_\beta|z\rangle^*\langle z|q_\alpha|w\rangle^*\Big)$$
$$+\Big(\sum_{fy}\frac{V^\alpha_{af}V^\alpha_{fc}}{E_c + E_w - E_f - E_y + i0^+}\langle v|q_\alpha|y\rangle\langle y|q_\alpha|w\rangle\Big)$$
$$\cdot\Big(\sum_{gz}\frac{(V^\beta_{bg})^*(V^\beta_{gd})^*}{E_d + E_w - E_g - E_z - i0^+}\langle v|q_\beta|z\rangle^*\langle z|q_\beta|w\rangle^*\Big)\Big]\,\delta(E_a + E_v - E_c - E_w)\rho^{\rm eq}_{ww}\,. \tag{S28}$$

However, the latter term, $(\alpha,\gamma) = (\beta,\delta)$, often referred to as a reducible diagram, is among those classified as divergent in the work of Timm [29]. The regularized $\mathcal{T}$-matrix formalism adopted here deals with this by simply removing it

[29]. The discussion of the justification of this step is beyond the scope of the present work and we point the interested reader to the full discussion on divergences cancellation based on time convolutionless master equations by Timm [29]. We thus focus on

$$\sum_{vw}\sum_{\alpha\beta}\Big[\Big(\sum_{fy}\frac{V^{\alpha}_{af}V^{\beta}_{fc}}{E_c+E_w-E_f-E_y+i0^+}\langle v|q_\alpha|y\rangle\langle y|q_\beta|w\rangle\Big)$$
$$\cdot\Big(\sum_{gz}\frac{(V^{\alpha}_{bg})^*(V^{\beta}_{gd})^*}{E_d+E_w-E_g-E_z-i0^+}\langle v|q_\alpha|z\rangle^*\langle z|q_\beta|w\rangle^*\Big)$$
$$+\Big(\sum_{fy}\frac{V^{\alpha}_{af}V^{\beta}_{fc}}{E_c+E_w-E_f-E_y+i0^+}\langle v|q_\alpha|y\rangle\langle y|q_\beta|w\rangle\Big)$$
$$\cdot\Big(\sum_{gz}\frac{(V^{\beta}_{bg})^*(V^{\alpha}_{gd})^*}{E_d+E_w-E_g-E_z-i0^+}\langle v|q_\beta|z\rangle^*\langle z|q_\alpha|w\rangle^*\Big)\Big]\,\delta(E_a+E_v-E_c-E_w)\rho^{\mathrm{eq}}_{ww}\,. \tag{S29}$$

In addition, each distinct pair of phonons will contribute four terms. For $|w\rangle=|n_\alpha n_\beta\rangle$, then $|v\rangle$ must be $|n_\alpha\pm1,n_\beta\pm1\rangle$. Let us start from $|w\rangle=|n_\alpha n_\beta\rangle$ and $|v\rangle=|n_\alpha+1,n_\beta+1\rangle$, leading to the expression

$$\sum_{\alpha\beta}\Big[\Big(\sum_{f}\frac{V^{\alpha}_{af}V^{\beta}_{fc}}{E_c-E_f-\hbar\omega_\beta+i0^+}\sqrt{n_\alpha+1}\sqrt{n_\beta+1}\Big)$$
$$\cdot\Big(\sum_{g}\frac{(V^{\alpha}_{bg})^*(V^{\beta}_{gd})^*}{E_d-E_g-\hbar\omega_\beta-i0^+}\sqrt{n_\alpha+1}\sqrt{n_\beta+1}\Big)$$
$$+\Big(\sum_{f}\frac{V^{\alpha}_{af}V^{\beta}_{fc}}{E_c-E_f-\hbar\omega_\beta+i0^+}\sqrt{n_\alpha+1}\sqrt{n_\beta+1}\Big)$$
$$\cdot\Big(\sum_{g}\frac{(V^{\beta}_{bg})^*(V^{\alpha}_{gd})^*}{E_d-E_g-\hbar\omega_\alpha-i0^+}\sqrt{n_\beta+1}\sqrt{n_\alpha+1}\Big)\Big]\,\delta(E_a-E_c+\hbar\omega_\alpha+\hbar\omega_\beta)\rho^{\mathrm{eq}}_{ww}\,. \tag{S30}$$

or equivalently

$$\sum_{\alpha\beta}\Big[\Big(\sum_{f}\frac{V^{\alpha}_{af}V^{\beta}_{fc}}{E_c-E_f-\hbar\omega_\beta+i0^+}\Big)\cdot\Big(\sum_{g}\frac{(V^{\alpha}_{bg})^*(V^{\beta}_{gd})^*}{E_d-E_g-\hbar\omega_\beta-i0^+}\Big)$$
$$+\Big(\sum_{f}\frac{V^{\alpha}_{af}V^{\beta}_{fc}}{E_c-E_f-\hbar\omega_\beta+i0^+}\Big)\cdot\Big(\sum_{g}\frac{(V^{\beta}_{bg})^*(V^{\alpha}_{gd})^*}{E_d-E_g-\hbar\omega_\alpha-i0^+}\Big)\Big]$$
$$\cdot\,(n_\alpha+1)\,(n_\beta+1)\,\delta(E_a-E_c+\hbar\omega_\alpha+\hbar\omega_\beta)\,\rho^{\mathrm{eq}}_{ww}\,. \tag{S31}$$

Finally, rewriting this expression by summing only on inequivalent pairs of phonons, and averaging over the canonical

distribution of initial states $|w\rangle$, we get

$$\sum_w \mathcal{T}_{a(v)c(w)}\mathcal{T}^*_{b(v)d(w)}\delta(E_a+E_v-E_c-E_w)\rho^{\rm eq}_{ww} = \tag{S32}$$

$$\begin{aligned}\sum_{\alpha>\beta}\Big[&\Big(\sum_f \frac{V^\alpha_{af}V^\beta_{fc}}{E_c-E_f-\hbar\omega_\beta+i0^+}\Big)\cdot\Big(\sum_g \frac{(V^\alpha_{bg})^*(V^\beta_{gd})^*}{E_d-E_g-\hbar\omega_\beta-i0^+}\Big)\\ &+\Big(\sum_f \frac{V^\alpha_{af}V^\beta_{fc}}{E_c-E_f-\hbar\omega_\beta+i0^+}\Big)\cdot\Big(\sum_g \frac{(V^\beta_{bg})^*(V^\alpha_{gd})^*}{E_d-E_g-\hbar\omega_\alpha-i0^+}\Big)\\ &+\Big(\sum_f \frac{V^\beta_{af}V^\alpha_{fc}}{E_c-E_f-\hbar\omega_\alpha+i0^+}\Big)\cdot\Big(\sum_g \frac{(V^\alpha_{bg})^*(V^\beta_{gd})^*}{E_d-E_g-\hbar\omega_\beta-i0^+}\Big)\\ &+\Big(\sum_f \frac{V^\beta_{af}V^\alpha_{fc}}{E_c-E_f-\hbar\omega_\alpha+i0^+}\Big)\cdot\Big(\sum_g \frac{(V^\beta_{bg})^*(V^\alpha_{gd})^*}{E_d-E_g-\hbar\omega_\alpha-i0^+}\Big)\Big]\\ &\cdot(\bar n_\alpha+1)\,(\bar n_\beta+1)\,\delta(E_a-E_c+\hbar\omega_\alpha+\hbar\omega_\beta)\,.\end{aligned} \tag{S33}$$

This is only one term out of all the twelve possible ones appearing in the final expression, which arise from considering four final states $|v\rangle$ and the three terms of Eq. (S15). Following similar steps and considerations, we can derive the contribution of $|v\rangle = |n_\alpha-1, n_\beta-1\rangle$ to Eq. (S15), which reads

$$\sum_w \mathcal{T}_{a(v)c(w)}\mathcal{T}^*_{b(v)d(w)}\delta(E_a+E_v-E_c-E_w)\rho^{\rm eq}_{ww} = \tag{S34}$$

$$\begin{aligned}\sum_{\alpha>\beta}\Big[&\Big(\sum_f \frac{V^\alpha_{af}V^\beta_{fc}}{E_c-E_f+\hbar\omega_\beta+i0^+}\Big)\cdot\Big(\sum_g \frac{(V^\alpha_{bg})^*(V^\beta_{gd})^*}{E_d-E_g+\hbar\omega_\beta-i0^+}\Big)\\ &+\Big(\sum_f \frac{V^\alpha_{af}V^\beta_{fc}}{E_c-E_f+\hbar\omega_\beta+i0^+}\Big)\cdot\Big(\sum_g \frac{(V^\beta_{bg})^*(V^\alpha_{gd})^*}{E_d-E_g+\hbar\omega_\alpha-i0^+}\Big)\\ &+\Big(\sum_f \frac{V^\beta_{af}V^\alpha_{fc}}{E_c-E_f+\hbar\omega_\alpha+i0^+}\Big)\cdot\Big(\sum_g \frac{(V^\alpha_{bg})^*(V^\beta_{gd})^*}{E_d-E_g+\hbar\omega_\beta-i0^+}\Big)\\ &+\Big(\sum_f \frac{V^\beta_{af}V^\alpha_{fc}}{E_c-E_f+\hbar\omega_\alpha+i0^+}\Big)\cdot\Big(\sum_g \frac{(V^\beta_{bg})^*(V^\alpha_{gd})^*}{E_d-E_g+\hbar\omega_\alpha-i0^+}\Big)\Big]\\ &\cdot\bar n_\alpha\,\bar n_\beta\,\delta(E_a-E_c-\hbar\omega_\alpha-\hbar\omega_\beta)\,.\end{aligned} \tag{S35}$$

For the two absorption/emission processes, $|v\rangle = |n_\alpha+1, n_\beta-1\rangle$ and $|v\rangle = |n_\alpha-1, n_\beta+1\rangle$ we can derive

$$\sum_w \mathcal{T}_{a(v)c(w)}\mathcal{T}^*_{b(v)d(w)}\delta(E_a+E_v-E_c-E_w)\rho^{\rm eq}_{ww} = \tag{S36}$$

$$\begin{aligned}\sum_{\alpha>\beta}\Big[&\Big(\sum_f \frac{V^\alpha_{af}V^\beta_{fc}}{E_a-E_f-\hbar\omega_\beta+i0^+}\Big)\cdot\Big(\sum_g \frac{(V^\alpha_{bg})^*(V^\beta_{gd})^*}{E_b-E_g-\hbar\omega_\beta-i0^+}\Big)\\ &+\Big(\sum_f \frac{V^\alpha_{af}V^\beta_{fc}}{E_a-E_f-\hbar\omega_\beta+i0^+}\Big)\cdot\Big(\sum_g \frac{(V^\beta_{bg})^*(V^\alpha_{gd})^*}{E_b-E_g+\hbar\omega_\alpha-i0^+}\Big)\\ &+\Big(\sum_f \frac{V^\beta_{af}V^\alpha_{fc}}{E_a-E_f+\hbar\omega_\alpha+i0^+}\Big)\cdot\Big(\sum_g \frac{(V^\alpha_{bg})^*(V^\beta_{gd})^*}{E_b-E_g-\hbar\omega_\beta-i0^+}\Big)\\ &+\Big(\sum_f \frac{V^\beta_{af}V^\alpha_{fc}}{E_a-E_f+\hbar\omega_\alpha+i0^+}\Big)\cdot\Big(\sum_g \frac{(V^\beta_{bg})^*(V^\alpha_{gd})^*}{E_b-E_g+\hbar\omega_\alpha-i0^+}\Big)\Big]\\ &\cdot\bar n_\alpha\,(\bar n_\beta+1)\,\delta(E_a-E_c-\hbar\omega_\alpha+\hbar\omega_\beta)\,,\end{aligned} \tag{S37}$$

and

$$\sum_w \mathcal{T}_{a(v)c(w)}\mathcal{T}^*_{b(v)d(w)}\delta(E_a+E_v-E_c-E_w)\rho^{\rm eq}_{ww} = \tag{S38}$$

$$\begin{aligned}
\sum_{\alpha>\beta}\Big[&\Big(\sum_f \frac{V^\alpha_{af}V^\beta_{fc}}{E_c-E_f-\hbar\omega_\beta+i0^+}\Big)\cdot\Big(\sum_g \frac{(V^\alpha_{bg})^*(V^\beta_{gd})^*}{E_d-E_g-\hbar\omega_\beta-i0^+}\Big)\\
&+\Big(\sum_f \frac{V^\alpha_{af}V^\beta_{fc}}{E_c-E_f-\hbar\omega_\beta+i0^+}\Big)\cdot\Big(\sum_g \frac{(V^\beta_{bg})^*(V^\alpha_{gd})^*}{E_d-E_g-\hbar\omega_\alpha-i0^+}\Big)\\
&+\Big(\sum_f \frac{V^\beta_{af}V^\alpha_{fc}}{E_c-E_f+\hbar\omega_\alpha+i0^+}\Big)\cdot\Big(\sum_g \frac{(V^\alpha_{bg})^*(V^\beta_{gd})^*}{E_d-E_g-\hbar\omega_\beta-i0^+}\Big)\\
&+\Big(\sum_f \frac{V^\beta_{af}V^\alpha_{fc}}{E_c-E_f+\hbar\omega_\alpha+i0^+}\Big)\cdot\Big(\sum_g \frac{(V^\beta_{bg})^*(V^\alpha_{gd})^*}{E_d-E_g+\hbar\omega_\alpha-i0^+}\Big)\Big]\\
&\cdot(\bar{n}_\alpha+1)\,\bar{n}_\beta\,\delta(E_a-E_c+\hbar\omega_\alpha-\hbar\omega_\beta)\,,
\end{aligned}\tag{S39}$$

respectively.

Let us now turn our attention to the case $\alpha=\beta$. In this case, Eq. (S27) results in

$$\begin{aligned}
\sum_\alpha\Big[&\Big(\sum_{fy} \frac{V^\alpha_{af}V^\alpha_{fc}}{E_c+E_w-E_f-E_y+i0^+}\langle v|q_\alpha|y\rangle\langle y|q_\alpha|w\rangle\Big)\\
&\cdot\Big(\sum_{gz} \frac{(V^\alpha_{bg})^*(V^\alpha_{gd})^*}{E_d+E_w-E_g-E_z-i0^+}\langle v|q_\alpha|z\rangle^*\langle z|q_\alpha|w\rangle^*\Big)\Big]\,\delta(E_a+E_v-E_c-E_w)\rho^{\rm eq}_{ww}\,,
\end{aligned}\tag{S40}$$

which is non-zero for $|v\rangle=|n_\alpha\pm 2\rangle$ or $|v\rangle=|w\rangle$. For $|v\rangle=|n_\alpha+2\rangle$, we get

$$\sum_\alpha\Big[\Big(\sum_f \frac{V^\alpha_{af}V^\alpha_{fc}}{E_c-E_f-\hbar\omega_\alpha+i0^+}\Big)\cdot\Big(\sum_g \frac{(V^\alpha_{bg})^*(V^\alpha_{gd})^*}{E_d-E_g-\hbar\omega_\alpha-i0^+}\Big)\Big](n_\alpha+1)(n_\alpha+1)\,\delta(E_a-E_c+2\hbar\omega_\alpha)\,\rho^{\rm eq}_{ww}\,, \tag{S41}$$

which is identical to any of the four terms of Eq. (S33), once $\alpha$ is set equal to $\beta$. We can therefore absorb the latter, and similarly the case for $|v\rangle=|n_\alpha-2\rangle$, into the general expressions for double absorption and double emission by setting a $1/4$ rescaling coefficient to avoid double counting.

Lastly, the case $|v\rangle=|w\rangle$ for $\alpha=\beta$ gives rise to a term of the form

$$\begin{aligned}
\sum_\alpha\Big[&\Big(\sum_f \frac{V^\alpha_{af}V^\alpha_{fc}}{E_a-E_f-\hbar\omega_\alpha+i0^+}\Big)\cdot\Big(\sum_g \frac{(V^\alpha_{bg})^*(V^\alpha_{gd})^*}{E_b-E_g-\hbar\omega_\alpha-i0^+}\Big)\\
&+\Big(\sum_f \frac{V^\alpha_{af}V^\alpha_{fc}}{E_a-E_f-\hbar\omega_\alpha+i0^+}\Big)\cdot\Big(\sum_g \frac{(V^\alpha_{bg})^*(V^\alpha_{gd})^*}{E_b-E_g+\hbar\omega_\alpha-i0^+}\Big)\\
&+\Big(\sum_f \frac{V^\alpha_{af}V^\alpha_{fc}}{E_a-E_f+\hbar\omega_\alpha+i0^+}\Big)\cdot\Big(\sum_g \frac{(V^\alpha_{bg})^*(V^\beta_{gd})^*}{E_b-E_g-\hbar\omega_\alpha-i0^+}\Big)\\
&+\Big(\sum_f \frac{V^\alpha_{af}V^\alpha_{fc}}{E_a-E_f+\hbar\omega_\alpha+i0^+}\Big)\cdot\Big(\sum_g \frac{(V^\alpha_{bg})^*(V^\alpha_{gd})^*}{E_b-E_g+\hbar\omega_\alpha-i0^+}\Big)\Big]n_\alpha\,(n_\alpha+1)\,\delta(E_a-E_c)\,\rho^{\rm eq}_{ww}\,.
\end{aligned}\tag{S42}$$

Also in this case, this term can be reabsorbed in the general absorption/emission case by applying a factor $1/2$ to avoid double-counting due to the fact that it would appear in both Eq. (S37) and Eq. (S39).

Following analogous steps for the second term of Eq. (S15), we can write

$$-\frac{\delta_{bd}}{2}\sum_{w}\sum_{ex}\mathcal{T}^{*}_{e(x)a(w)}\mathcal{T}_{e(x)c(w)}\;\delta(E_e+E_x-E_c-E_w)\rho^{\mathrm{eq}}_{ww} =$$

$$-\frac{\delta_{bd}}{2}\sum_{w}\sum_{ex}\sum_{\alpha\beta}\sum_{\gamma\delta}\Big(\sum_{fy}\frac{(V^{\alpha}_{ef})^{*}(V^{\beta}_{fa})^{*}}{E_a+E_w-E_f-E_y-i0^{+}}\langle x|q_\alpha|y\rangle^{*}\langle y|q_\beta|w\rangle^{*}\Big)$$

$$\cdot\Big(\sum_{gz}\frac{V^{\gamma}_{eg}V^{\delta}_{gc}}{E_c+E_w-E_g-E_z+i0^{+}}\langle x|q_\gamma|z\rangle\langle z|q_\delta|w\rangle\Big)\;\delta(E_e+E_x-E_c-E_w)\rho^{\mathrm{eq}}_{ww}\,. \tag{S43}$$

Then consider the non-zero contributions $(\alpha,\beta)=(\gamma,\delta)$ and $(\alpha,\beta)=(\delta,\gamma)$ for $\alpha\neq\beta$

$$-\frac{\delta_{bd}}{2}\sum_{w}\sum_{ex}\sum_{\alpha\beta}\Big[\Big(\sum_{fy}\frac{(V^{\alpha}_{ef})^{*}(V^{\beta}_{fa})^{*}}{E_a+E_w-E_f-E_y-i0^{+}}\langle x|q_\alpha|y\rangle^{*}\langle y|q_\beta|w\rangle^{*}\Big)$$

$$\cdot\Big(\sum_{gz}\frac{V^{\alpha}_{eg}V^{\beta}_{gc}}{E_c+E_w-E_g-E_z+i0^{+}}\langle x|q_\alpha|z\rangle\langle z|q_\beta|w\rangle\Big) \tag{S44}$$

$$+\Big(\sum_{fy}\frac{(V^{\alpha}_{ef})^{*}(V^{\beta}_{fa})^{*}}{E_a+E_w-E_f-E_y-i0^{+}}\langle x|q_\alpha|y\rangle^{*}\langle y|q_\beta|w\rangle^{*}\Big)$$

$$\cdot\Big(\sum_{gz}\frac{V^{\beta}_{eg}V^{\alpha}_{gc}}{E_c+E_w-E_g-E_z+i0^{+}}\langle x|q_\beta|z\rangle\langle z|q_\alpha|w\rangle\Big)\Big]\;\delta(E_e+E_x-E_c-E_w)\rho^{\mathrm{eq}}_{ww}\,. \tag{S45}$$

Similarly to all other two-phonon contributions, we will get non-zero terms for $|x\rangle=|n_\alpha\pm1,n_\beta\pm1\rangle$. For the double absorption case, $|x\rangle=|n_\alpha+1,n_\beta+1\rangle$, we get

$$-\frac{\delta_{bd}}{2}\sum_{w}\sum_{ex}\mathcal{T}^{*}_{e(x)a(w)}\mathcal{T}_{e(x)c(w)}\;\delta(E_e+E_x-E_c-E_w)\rho^{\mathrm{eq}}_{ww} =$$

$$-\frac{\delta_{bd}}{2}\sum_{e}\sum_{\alpha>\beta}\Big[\Big(\sum_{f}\frac{(V^{\alpha}_{ef})^{*}(V^{\beta}_{fa})^{*}}{E_a-E_f-\hbar\omega_\beta-i0^{+}}\Big)\cdot\Big(\sum_{g}\frac{V^{\alpha}_{eg}V^{\beta}_{gc}}{E_c-E_g-\hbar\omega_\beta+i0^{+}}\Big) \tag{S46}$$

$$+\Big(\sum_{f}\frac{(V^{\alpha}_{ef})^{*}(V^{\beta}_{fa})^{*}}{E_a-E_f-\hbar\omega_\beta-i0^{+}}\Big)\cdot\Big(\sum_{g}\frac{V^{\beta}_{eg}V^{\alpha}_{gc}}{E_c-E_g-\hbar\omega_\alpha+i0^{+}}\Big) \tag{S47}$$

$$+\Big(\sum_{f}\frac{(V^{\beta}_{ef})^{*}V^{\alpha}_{fa})^{*}}{E_a-E_f-\hbar\omega_\alpha-i0^{+}}\Big)\cdot\Big(\sum_{g}\frac{V^{\beta}_{eg}V^{\alpha}_{gc}}{E_c-E_g-\hbar\omega_\alpha+i0^{+}}\Big) \tag{S48}$$

$$+\Big(\sum_{f}\frac{(V^{\beta}_{ef})^{*}(V^{\alpha}_{fa})^{*}}{E_a-E_f-\hbar\omega_\alpha-i0^{+}}\Big)\cdot\Big(\sum_{g}\frac{V^{\alpha}_{eg}V^{\beta}_{gc}}{E_c-E_g-\hbar\omega_\beta+i0^{+}}\Big)\Big] \tag{S49}$$

$$(\bar{n}_\alpha+1)(\bar{n}_\beta+1)\delta(E_e-E_c+\hbar\omega_\alpha+\hbar\omega_\beta)\,. \tag{S50}$$

The $\alpha=\beta$ case is handled identically as for the first term of (S15).

We can ultimately write the full expression for the fourth-order contribution to $R_{ab,cd}$, which accounts for four possible two-phonon processes

$$R^{(4)}_{ab,cd} = R^{++}_{ab,cd} + R^{--}_{ab,cd} + R^{+-}_{ab,cd} + R^{-+}_{ab,cd}\,. \tag{S51}$$

By introducing the notation $A_{\alpha\beta} = (1 - \delta_{\alpha\beta}3/4)$, $B_{\alpha\beta} = (1 - \delta_{\alpha\beta}1/2)$

$$T^{\alpha\beta,\pm}_{ab} = \sum_f \frac{V^{\alpha}_{af} V^{\beta}_{fb}}{E_f - E_b \pm \hbar\omega_\beta - i0^+}\,, \tag{S52}$$

and

$$G^{(4)}_{++}(\omega_{ab}, \omega_\alpha, \omega_\beta) = (\bar{n}_\alpha + 1)\,(\bar{n}_\beta + 1)\,\delta(\omega_{ab} + \omega_\alpha + \omega_\beta)\,, \tag{S53}$$

$$G^{(4)}_{--}(\omega_{ab}, \omega_\alpha, \omega_\beta) = \bar{n}_\alpha\,\bar{n}_\beta\,\delta(\omega_{ab} - \omega_\alpha - \omega_\beta)\,, \tag{S54}$$

$$G^{(4)}_{+-}(\omega_{ab}, \omega_\alpha, \omega_\beta) = (\bar{n}_\alpha + 1)\,\bar{n}_\beta\,\delta(\omega_{ab} + \omega_\alpha - \omega_\beta)\,, \tag{S55}$$

$$G^{(4)}_{-+}(\omega_{ab}, \omega_\alpha, \omega_\beta) = \bar{n}_\alpha\,(\bar{n}_\beta + 1)\,\delta(\omega_{ab} - \omega_\alpha + \omega_\beta)\,, \tag{S56}$$

we can then finally write all four terms in a compact form as

$$R^{++}_{ab,cd} = \frac{2\pi}{\hbar^2}\sum_{\alpha\geq\beta} A_{\alpha\beta}\Big[T^{\alpha\beta,+}_{ac}(T^{\alpha\beta,+}_{bd})^* + T^{\alpha\beta,+}_{ac}(T^{\beta\alpha,+}_{bd})^* + T^{\beta\alpha,+}_{ac}(T^{\alpha\beta,+}_{bd})^* + T^{\beta\alpha,+}_{ac}(T^{\beta\alpha,+}_{bd})^*\Big] G^{(4)}_{++}(\omega_{ac}, \omega_\alpha, \omega_\beta) \tag{S57}$$

$$-\frac{2\pi}{\hbar^2}\frac{\delta_{bd}}{2}\sum_e\sum_{\alpha\geq\beta} A_{\alpha\beta}\Big[T^{\alpha\beta,+}_{ec}(T^{\alpha\beta,+}_{ea})^* + T^{\alpha\beta,+}_{ec}(T^{\beta\alpha,+}_{ea})^* + T^{\beta\alpha,+}_{ec}(T^{\alpha\beta,+}_{ea})^* + T^{\beta\alpha,+}_{ec}(T^{\beta\alpha,+}_{ea})^*\Big] G^{(4)}_{++}(\omega_{ec}, \omega_\alpha, \omega_\beta) \tag{S58}$$

$$-\frac{2\pi}{\hbar^2}\frac{\delta_{ac}}{2}\sum_e\sum_{\alpha\geq\beta} A_{\alpha\beta}\Big[T^{\alpha\beta,+}_{eb}(T^{\alpha\beta,+}_{ed})^* + T^{\alpha\beta,+}_{eb}(T^{\beta\alpha,+}_{ed})^* + T^{\beta\alpha,+}_{eb}(T^{\alpha\beta,+}_{ed})^* + T^{\beta\alpha,+}_{eb}(T^{\beta\alpha,+}_{ed})^*\Big] G^{(4)}_{++}(\omega_{ed}, \omega_\alpha, \omega_\beta)\,, \tag{S59}$$

$$R^{--}_{ab,cd} = \frac{2\pi}{\hbar^2}\sum_{\alpha\geq\beta} A_{\alpha\beta}\Big[T^{\alpha\beta,-}_{ac}(T^{\alpha\beta,-}_{bd})^* + T^{\alpha\beta,-}_{ac}(T^{\beta\alpha,-}_{bd})^* + T^{\beta\alpha,-}_{ac}(T^{\alpha\beta,-}_{bd})^* + T^{\beta\alpha,-}_{ac}(T^{\beta\alpha,-}_{bd})^*\Big] G^{(4)}_{--}(\omega_{ac}, \omega_\alpha, \omega_\beta) \tag{S60}$$

$$-\frac{2\pi}{\hbar^2}\frac{\delta_{bd}}{2}\sum_e\sum_{\alpha\geq\beta} A_{\alpha\beta}\Big[T^{\alpha\beta,-}_{ec}(T^{\alpha\beta,-}_{ea})^* + T^{\alpha\beta,-}_{ec}(T^{\beta\alpha,-}_{ea})^* + T^{\beta\alpha,-}_{ec}(T^{\alpha\beta,-}_{ea})^* + T^{\beta\alpha,-}_{ec}(T^{\beta\alpha,-}_{ea})^*\Big] G^{(4)}_{--}(\omega_{ec}, \omega_\alpha, \omega_\beta) \tag{S61}$$

$$-\frac{2\pi}{\hbar^2}\frac{\delta_{ac}}{2}\sum_e\sum_{\alpha\geq\beta} A_{\alpha\beta}\Big[T^{\alpha\beta,-}_{eb}(T^{\alpha\beta,-}_{ed})^* + T^{\alpha\beta,-}_{eb}(T^{\beta\alpha,+}_{ed})^* + T^{\beta\alpha,-}_{eb}(T^{\alpha\beta,-}_{ed})^* + T^{\beta\alpha,-}_{eb}(T^{\beta\alpha,-}_{ed})^*\Big] G^{(4)}_{--}(\omega_{ed}, \omega_\alpha, \omega_\beta)\,, \tag{S62}$$

$$R^{+-}_{ab,cd} = \frac{2\pi}{\hbar^2}\sum_{\alpha\geq\beta} B_{\alpha\beta}\Big[T^{\alpha\beta,-}_{ac}(T^{\alpha\beta,-}_{bd})^* + T^{\alpha\beta,-}_{ac}(T^{\beta\alpha,+}_{bd})^* + T^{\beta\alpha,+}_{ac}(T^{\alpha\beta,-}_{bd})^* + T^{\beta\alpha,+}_{ac}(T^{\beta\alpha,+}_{bd})^*\Big] G^{(4)}_{+-}(\omega_{ac}, \omega_\alpha, \omega_\beta) \tag{S63}$$

$$-\frac{2\pi}{\hbar^2}\frac{\delta_{bd}}{2}\sum_e\sum_{\alpha\geq\beta} B_{\alpha\beta}\Big[T^{\alpha\beta,-}_{ec}(T^{\alpha\beta,-}_{ea})^* + T^{\alpha\beta,-}_{ec}(T^{\beta\alpha,+}_{ea})^* + T^{\beta\alpha,+}_{ec}(T^{\alpha\beta,-}_{ea})^* + T^{\beta\alpha,+}_{ec}(T^{\beta\alpha,+}_{ea})^*\Big] G^{(4)}_{+-}(\omega_{ec}, \omega_\alpha, \omega_\beta) \tag{S64}$$

$$-\frac{2\pi}{\hbar^2}\frac{\delta_{ac}}{2}\sum_e\sum_{\alpha\geq\beta} B_{\alpha\beta}\Big[T^{\alpha\beta,-}_{eb}(T^{\alpha\beta,-}_{ed})^* + T^{\alpha\beta,-}_{eb}(T^{\beta\alpha,+}_{ed})^* + T^{\beta\alpha,+}_{eb}(T^{\alpha\beta,-}_{ed})^* + T^{\beta\alpha,+}_{eb}(T^{\beta\alpha,+}_{ed})^*\Big] G^{(4)}_{+-}(\omega_{ed}, \omega_\alpha, \omega_\beta)\,, \tag{S65}$$

$$R^{-+}_{ab,cd} = \frac{2\pi}{\hbar^2}\sum_{\alpha\geq\beta} B_{\alpha\beta}\Big[T^{\alpha\beta,+}_{ac}(T^{\alpha\beta,+}_{bd})^* + T^{\alpha\beta,+}_{ac}(T^{\beta\alpha,-}_{bd})^* + T^{\beta\alpha,-}_{ac}(T^{\alpha\beta,+}_{bd})^* + T^{\beta\alpha,-}_{ac}(T^{\beta\alpha,-}_{bd})^*\Big] G^{(4)}_{-+}(\omega_{ac}, \omega_\alpha, \omega_\beta) \tag{S66}$$

$$-\frac{2\pi}{\hbar^2}\frac{\delta_{bd}}{2}\sum_e\sum_{\alpha\geq\beta} B_{\alpha\beta}\Big[T^{\alpha\beta,+}_{ec}(T^{\alpha\beta,+}_{ea})^* + T^{\alpha\beta,+}_{ec}(T^{\beta\alpha,-}_{ea})^* + T^{\beta\alpha,-}_{ec}(T^{\alpha\beta,+}_{ea})^* + T^{\beta\alpha,-}_{ec}(T^{\beta\alpha,-}_{ea})^*\Big] G^{(4)}_{-+}(\omega_{ec}, \omega_\alpha, \omega_\beta) \tag{S67}$$

$$-\frac{2\pi}{\hbar^2}\frac{\delta_{ac}}{2}\sum_e\sum_{\alpha\geq\beta} B_{\alpha\beta}\Big[T^{\alpha\beta,+}_{eb}(T^{\alpha\beta,+}_{ed})^* + T^{\alpha\beta,+}_{eb}(T^{\beta\alpha,-}_{ed})^* + T^{\beta\alpha,-}_{eb}(T^{\alpha\beta,+}_{ed})^* + T^{\beta\alpha,-}_{eb}(T^{\beta\alpha,-}_{ed})^*\Big] G^{(4)}_{-+}(\omega_{ed}, \omega_\alpha, \omega_\beta)\,. \tag{S68}$$

Once these equations are evaluated for transition among population terms, i.e. $R_{aa,bb}$, the same result of Ref. [18] is recovered.

**Quantum master equations in the Lindblad form**

Finally, we establish the connection between the expressions just derived and the general form for quantum master equations describing Markov processes. According to the theorem by Lindblad, it is always possible to express the time evolution

$$\dot{\rho}_{ab} = \sum_{\kappa} \gamma_{\kappa} \left[ L^{\kappa}_{ac} (L^{\kappa}_{bd})^{*} - \frac{\delta_{db}}{2} \sum_{e} (L^{\kappa}_{ea})^{*} L_{ec} - \frac{\delta_{ac}}{2} \sum_{e} (L^{\kappa}_{ed})^{\kappa} L_{eb} \right] , \tag{S69}$$

where the operators $\hat{L}^{\kappa}$ are called jump operators and describe the action of the Markovian bath on the dynamics of the reduced density matrix through $\kappa$ contributions.

We can use this expression also to define the time spin relaxation and decoherence constants, $T_1$ and $T_2$, respectively. $T_1$ represents the lifetime of a given population state, $a$, and it is given by the element

$$\frac{1}{T_1} = -R_{aa,aa} = \sum_{\kappa} \gamma^{\kappa} \sum_{e \neq a} (L^{\kappa}_{ea})^{*} L^{\kappa}_{ea} = \sum_{e \neq a} R_{ee,aa} , \tag{S70}$$

where we can recognize that it is the sum of the probability to make a transition for $a$ to any other state of the system.

The decoherence time $T_2$ for the superposition of states $a$ and $b$ is instead given by the term

$$\frac{1}{T_2} = -R_{ab,ab} = \sum_{\kappa} \gamma^{\kappa} \Big[ -(L^{\kappa}_{aa})^{*} L^{\kappa}_{bb} + \frac{1}{2} \sum_{e} (L^{\kappa}_{ea})^{*} L^{\kappa}_{ea} + \frac{1}{2} \sum_{e} (L^{\kappa}_{eb})^{*} L^{\kappa}_{eb} \Big] . \tag{S71}$$

By inspecting Eq. (S71) we notice that the definition of $T_1$ for the states $a$ and $b$ appears in the expression of $T_2$. If $R_{aa,aa}$ and $R_{bb,bb}$ are similar in magnitude, as it would be for the two Kramers states discussed in the main text, then we can rewrite Eq. (S71) in terms of lifetime and pure-dephasing contributions, namely

$$\frac{1}{T_2} = \frac{1}{2T_1} + \frac{1}{T_2^{*}} , \tag{S72}$$

where

$$\frac{1}{2T_1} = \sum_{\kappa} \gamma^{\kappa} \Big[ \frac{1}{2} \sum_{e \neq a} (L^{\kappa}_{ea})^{*} L^{\kappa}_{ea} + \frac{1}{2} \sum_{e \neq a} (L^{\kappa}_{eb})^{*} L^{\kappa}_{eb} \Big] , \tag{S73}$$

and

$$\frac{1}{T_2^{*}} = \sum_{\kappa} \gamma^{\kappa} \Big[ -(L^{\kappa}_{aa})^{*} L^{\kappa}_{bb} + \frac{1}{2} (L^{\kappa}_{aa})^{*} L^{\kappa}_{aa} + \frac{1}{2} (L^{\kappa}_{bb})^{*} L^{\kappa}_{bb} \Big] . \tag{S74}$$

Finally, we note that it is evident that our expressions have a Lindbladian form and that the jump operators take different definitions at different orders.

At the second order, the summation on $\kappa$ runs on the single phonon excitations $\alpha$ and the jump operator reads

$$\sqrt{\gamma_{\kappa}} L^{\kappa}_{ac} = \frac{\sqrt{2\pi \, G^{(2)}(\omega, \omega_{\alpha})}}{\hbar} V^{\alpha}_{ac} , \tag{S75}$$

At the fourth order, the bath contributions $\kappa$ instead run over distinct phonon pairs ($\alpha$, $\beta$) and possible processes ($++$, $--$, $-+$, $+-$). The jump operator for the absorption/emission process reads

$$\sqrt{\gamma_{\kappa}} L^{\kappa}_{ac} = \frac{\sqrt{2\pi \, G^{(4)}_{-+}(\omega, \omega_{\alpha}, \omega_{\beta})}}{\hbar} \left[ T^{\alpha\beta,+}_{ac} + T^{\beta\alpha,-}_{ac} \right] , \tag{S76}$$

and analogously for the other three contributions.

## Supplementary Figures

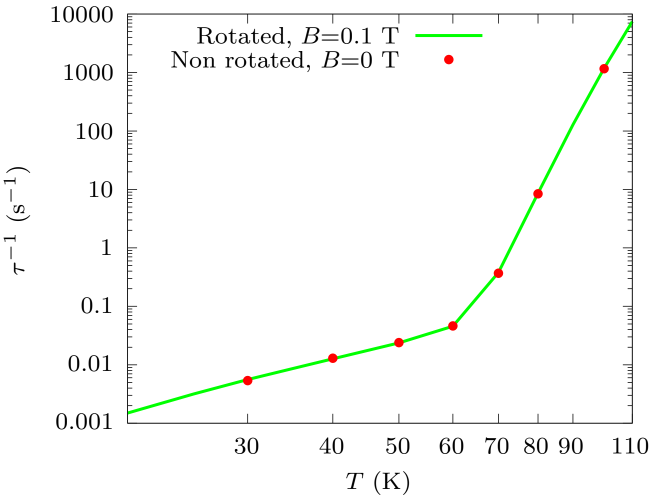


FIG. 1. **Rotational invariance of results.** Simulations of magnetic relaxation are performed for both the arbitrary crystal orientation in zero magnetic field and by orienting the molecule's easy axis along $z$ and by applying a magnetic field along the same direction. The agreement between the two confirms that the protocol proposed in Ref. [18] provides results consistent with the correct treatment of the full secular contributions of $R^{(4)}_{ab,cd}$.
